# Is it necessary to remove small fibular ossicles during an arthroscopic modified Broström operation for chronic lateral ankle instability?

**DOI:** 10.1186/s12891-025-08546-7

**Published:** 2025-04-21

**Authors:** Sung Hwan Kim, Sang Heon Lee, Seung Jin Choi, Young Koo Lee

**Affiliations:** Department of Orthopaedic Surgery, Soonchunhyang University Bucheon Hospital, Soonchunhyang University College of Medicine, 170 Jomaru-ro, Bucheon, 14584 Korea

**Keywords:** Ankle injury, Arthroscopy, Modified Brostrom operation, Ossicle, Sprains and strains

## Abstract

**Background:**

Ankle sprains are the most frequent musculoskeletal injury in sports. Patients reporting pain at the lateral malleolus tip following ankle sprains or sports activities frequently have separated ossicles, referred to as an os subfibulare (OSF). Commonly, small ossicles accompanied by chronic lateral ankle instability (CLAI) are treated with ossicle resection combined with the modified Broström operation (MBO). We compared the clinical and radiological results between groups in which a small OSF was or was not removed.

**Methods:**

We retrospectively enrolled all patients with a small OSF who underwent arthroscopic MBO by one surgeon in one institution between 2015 and 2022. The study included skeletally mature patients who had an OSF among those who had MBO surgery and follow-up for at least 1 year. An ossicle was defined as small if the longitudinal diameter was < 5 mm on an anteroposterior plain radiograph.

**Results:**

There were no significant differences between the groups preoperatively or 6 or 12 months postoperatively. The radiographic findings did not differ significantly between groups.

**Conclusions:**

When performing arthroscopic MBO on patients with CLAI, OSF ≤ 5 mm removal did not alter clinical or radiological outcomes, suggesting that excision may not be needed in asymptomatic patients. Considering the risks of the removal process, leaving it alone may be a treatment option.

**Clinical trial number:**

Not applicable.

**Supplementary Information:**

The online version contains supplementary material available at 10.1186/s12891-025-08546-7.

## Background

Ankle sprains are the most frequent musculoskeletal injury in sports [1]. Recurrent sprains and repeated instances of the ankle giving way often lead to chronic lateral ankle instability (CLAI) [2]. Several surgical techniques have been introduced for treating CLAI [3], including the modified Broström operation (MBO), which involves repairing the anterior talofibular ligaments (ATFL) and calcaneofibular ligaments (CFL) and augmenting the extensor retinaculum, has been used extensively with satisfactory outcomes [4, 5]. Recently, all-inside arthroscopic MBO has been developed with good to excellent results [6]. 

Patients reporting pain at the lateral malleolus tip following ankle sprains or sports activities frequently have separated ossicles, referred to as an os subfibulare (OSF) [7–9]. A subfibular ossicle is commonly described as a small, well-corticated separate ossicle located at the distal end of the fibula. There are two hypotheses on the origin of such ossicles: they arise from an accessory ossification center due to abnormal ossification or they result from an avulsion fracture in which either a cartilaginous or osseous fragment is detached from the tip of the fibula [10]. 

A separated ossicle is not always symptomatic; however, they may become symptomatic during trauma or overuse, such as during exercise, necessitating treatment [11]. It is believed that lateral ankle pain arises from traction stress on the ossicle from the attached ligament or from surrounding synovitis and hypertrophic soft tissue impingement [12, 13]. Hasegawa et al. classified OSF less than 5 mm as small size [13]. Commonly, small ossicles accompanied by CLAI are treated with ossicle resection combined with the MBO [14], although some studies have reported less favorable outcomes following this approach [2]. Other studies have reported similar clinical outcomes regardless of the presence of an OSF [7, 15, 16]. These ossicles are typically embedded within the fibers of the ATFL and are often extracted before reconstructing the lateral ligaments [13]. However, due to concerns about potential damage during removal, particularly since the OSF is embedded in the ATFL, some surgeons have opted not to remove an OSF, especially when it is small and challenging to extract, and the patient has no tenderness of the lateral ankle. In outpatient follow-up, the clinical and radiological outcomes were comparable to those of patients in whom the OSF was removed [16]. This led to the hypothesis that if the OSF is small, there might be no need to remove it despite potential soft tissue damage. To test this hypothesis, we compared the clinical and radiological results between groups in which a small OSF was or was not removed.

## Methods

The study was conducted in accordance with the Declaration of Helsinki, and approved by the Institutional Review Board (IRB) and Human Research Ethics Committee (IRB No. 2024-04-005, 04 April 2024).

### Patient selection

We retrospectively enrolled all patients with a small OSF who underwent arthroscopic MBO by a single surgeon at our hospital between 2015 and 2022. The study included skeletally mature patients who had an OSF among those who had MBO surgery and follow-up for at least 1 year. We excluded patients with an osteochondral lesion, neurological impairment of the lower limbs, fracture, or inflammatory diseases of the ankle as all these conditions can affect patient outcomes.

The size of the OSF and ligament injury of the ankle were evaluated from plain radiographs and magnetic resonance imaging. All radiological findings were confirmed at intraoperative arthroscopy. The results were used to select patients for study inclusion. An ossicle was defined as small if the longitudinal diameter was < 5 mm on an anteroposterior plain radiograph [8, 13]. 

### Surgical management

We perform arthroscopic MBO using the all-inside technique [6]. The surgical procedure involved reconstruction of the ATFL by reinforcing the inferior extensor retinaculum. After trimming any fibers of the distal tibiofibular ligament by shaver and vapor, synovial tissue and periosteum were then carefully removed with a shaver and radiofrequency below the anterior tibiofibular ligament. By removing the bony tissue using motorized burr, bleeding bone surface was revealed. In the case where OSF was removed, OSF was removed during this step. Because the size of the OSF was small, in almost all cases, the OSF was removed through the portal simply using arthroscopic forcep. A drill hole was created at a right angle to the anterior surface of the fibula, and the anchor was inserted through the anterolateral portal. A single absorbable Bio-Suture Tak anchor was tagged with two sutures (3.0 mm; FiberWire and TigerWire; Arthrex, Naples, FL, USA). An additional anteroinferior portal was made around the sinus tarsi area to tighten the anchor wire. Furthermore, a far-lateral portal was established distal to the anterior fibula. Holding one end of the anchor sutures, penetrator was passed through the anterolateral portal into the accessory anteroinferior portal intra-articularly. The other end of each suture was then pulled through the far-lateral portal using the penetrator. To pull the sutures under the skin, a suture retriever was inserted through the far-lateral portal into the accessory anteroinferior portal. Keeping the patient’s foot positioned dorsiflexion and eversion, the knot was finally tightened.

### Radiographic evaluation

**Fig. 1:**
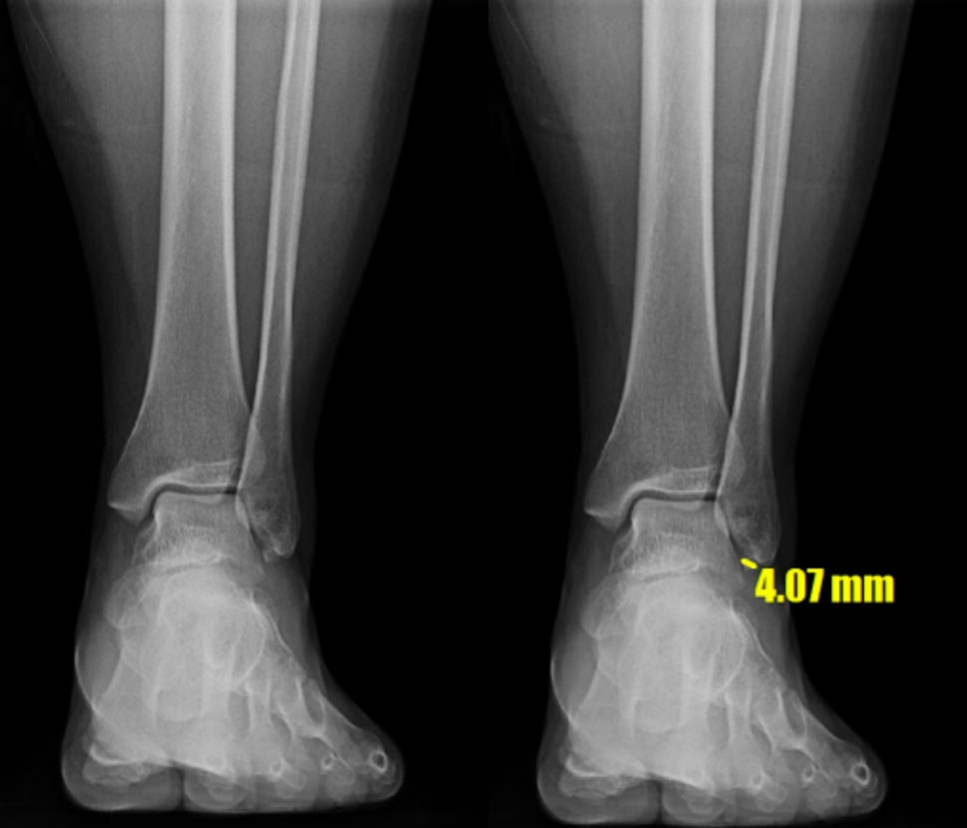
Technique for measuring size of ossicle

**Fig. 2:**
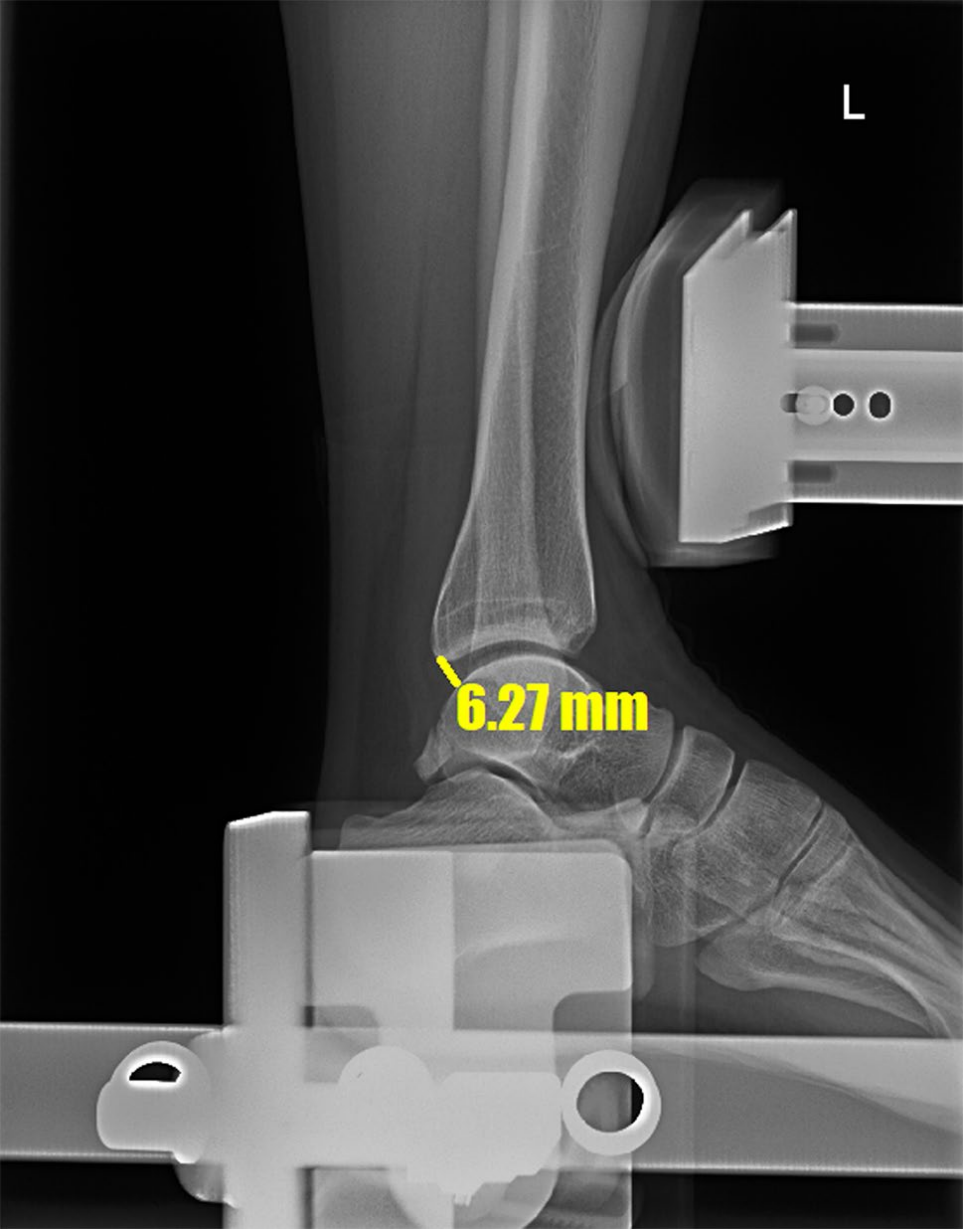
Technique for measuring ADT

**Fig. 3:**
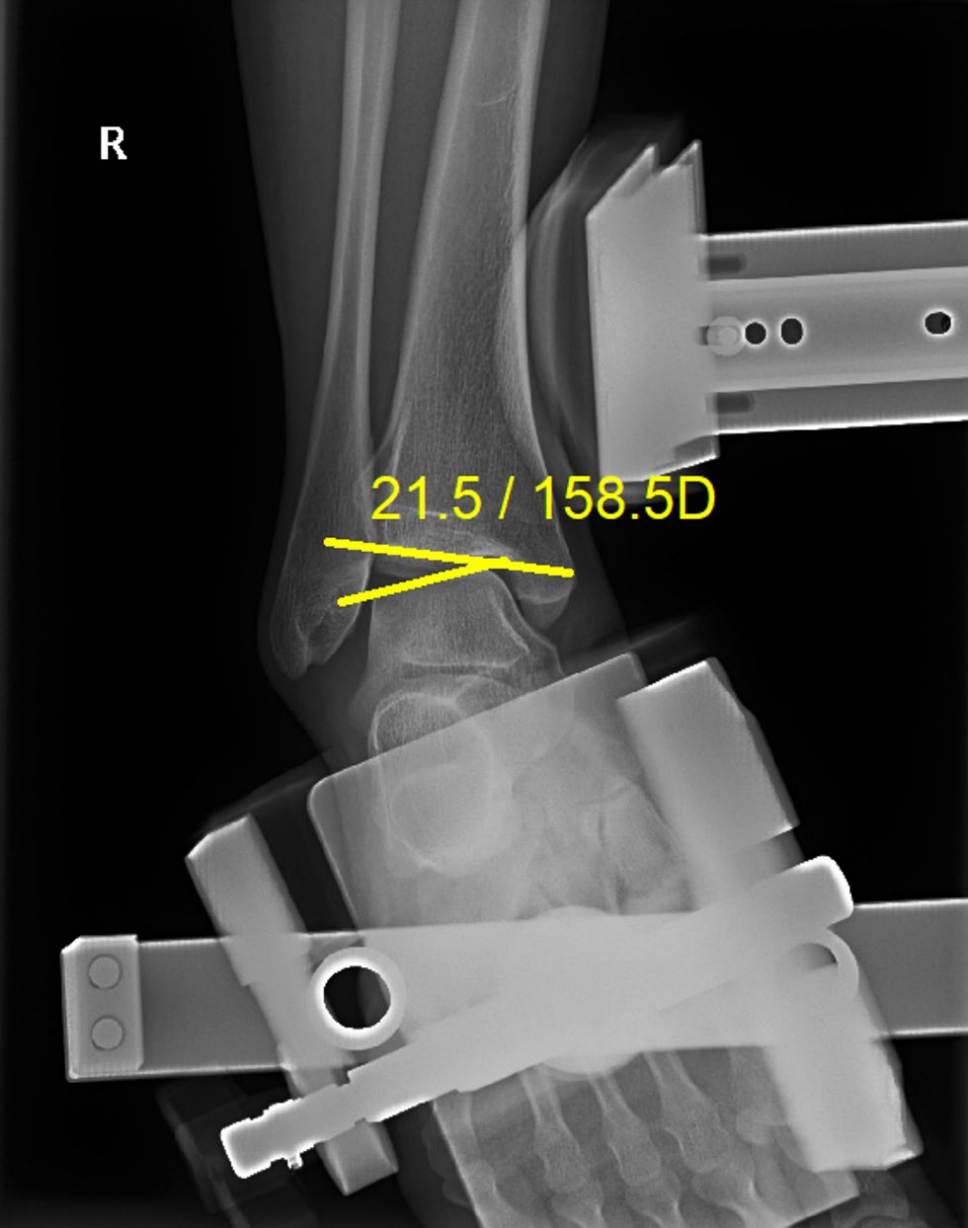
Technique for measuring TTA

The OSF were sized using the longest diameter on ankle radiographs (Fig. 1) [7]. Stress radiographs were taken preoperatively, as well as at 6, 12, and 24 months postoperatively and final follow up in outpatient by using a Telometer (Daeseung, Seoul, Korea). Standard measurements were made while performing the anterior drawer test (ADT) and talar tilt angle (TTA) tests. The stress radiographs were obtained with the foot positioned at approximately 10° of plantarflexion. Using lateral radiographs with keeping the patient’s leg slightly internally rotated, the ADT was measured. During the stress radiographs, a 150 N force was applied to the ankles in both the ADT and talar tilt planes, while the patient was made to relax their leg muscles [17, 18]. The procedures were well-tolerated and did not cause significant discomfort or pain. All radiographic examinations were performed by a senior radiologist with expertise in musculoskeletal imaging. Three orthopedic surgeons (a third-year resident, fellow, and professor) assessed the stress radiograph measurements using a picture archiving and communication system (PACS) without knowing the operative treatment. To evaluate intra- and interobserver reliability, the orthopedic surgeons repeated the measurements three times using the PACS at two-week intervals. The width of the posterior area of the ankle joint was referred to as the ADT. The reference points used for this measurement were the posterior border of the tibia and the proximal posterior articular surface of the talus. The shortest distance between these points was measured and recorded as the ADT (Fig. 2). When the observer drew a line connecting the reference points on the tibia and talus, this line formed a 90° angle with the tangent to the talar articular surface. The TTA was defined as the angle between the articular surfaces of the distal tibia and talus on anteroposterior stress radiographs (Fig. 3) [4]. 

### Clinical outcomes

We used the Foot and Ankle Outcome Score (FAOS), a validated, reliable, disease-specific, patient-reported outcome measure, to assess functional limitations in daily activities and symptoms among patients with foot and ankle disorders. The FAOS has five domains: symptoms, pain, activities of daily living, sports and recreation, and quality of life. We also adopted the American Orthopaedic Foot and Ankle Society (AOFAS) clinical rating system. Additionally, subjective pain was evaluated using a visual analog scale (VAS).

### Statistical methods

Continuous variables were evaluated using the Shapiro–Wilk test, which revealed that the data were not normally distributed; thus, nonparametric statistical tests were used for comparisons. Continuous variables are presented as medians (25th and 75th percentiles), whereas categorical variables are presented as frequencies (percentages). Variables were compared between independent groups using the Wilcoxon rank-sum test and Pearson’s χ^2^ test, as appropriate. All tests were two-tailed, and *p*-values less than 0.05 were considered statistically significant.

As this study involved a retrospective analysis of all patients in a registry, no a priori power calculation was conducted. Consequently, statistical power was verified through two-sided two-sample equal-variance *t*-tests. Using groupwise proportions and setting the number of patients to 44, the current study had a power of 92.9% to detect meaningful clinical differences in the FAOS, AOFAS, and VAS. The statistical analyses were performed using Rex ver. 3.03 (RexSoft, Seoul, Korea).

## Results

**Fig. 4:**
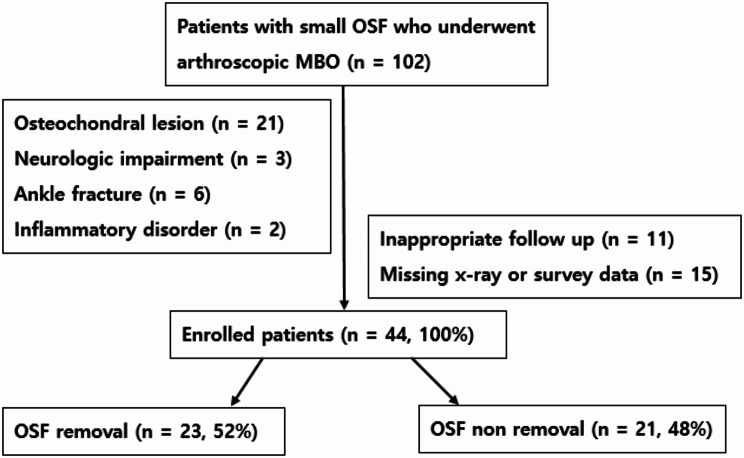
Cohort flowchart. OSF: os subfibulare, MBO: modified Broström operation

Between 2015 and 2022, 102 patients with a small OSF underwent arthroscopic MBO surgery by a single surgeon at our hospital. Of these, 32 patients were excluded from the study because of an osteochondral lesion, ankle fracture, neurologic impairment of the lower limbs, or inflammatory diseases of the ankle. Of the remaining patients, 59 were followed as outpatients for ≥ 1 year and had available clinical outcome data; 15 were excluded because of a missing postoperative stress X-ray or survey data. Finally, the study included 44 patients, of whom 23 underwent OSF removal (Fig. 4). The mean ossicle size was 3.86 mm. The mean patient age was 32.5 years. Table 1 summarizes the demographics of the patients in both study groups. The sex ratio, age, and side of injury did not differ significantly between the groups.

**Table 1:** Demographic characteristics of the patients in the OSF removed and not removed groups

Variable	Total (*n* = 44)	OSF removed (*n* = 23)	OSF not removed (*n* = 21)	*p*-value
Age (yr)	32.5 (23–45.25)	25 (22.5–40)	38 (27–51)	0.0523
Sex				0.9555
Female	18 (40.91)	10 (43.48)	8 (38.1)	
Male	26 (59.09)	13 (56.52)	13 (61.9)	
Side of injury				0.5768
Right	18 (40.91)	8 (34.78)	10 (47.62)	
Left	26 (59.09)	15 (65.22)	11 (52.38)	

Values are presented as mean (range) or number (%)

OSF: os subfibulare

Table 2 compares the clinical outcomes between the groups preoperatively and 6 and 12 months postoperatively. The AOFAS and FAOS had improved at the final follow-up in both groups. There were no significant differences between the groups preoperatively or 6 or 12 months postoperatively. The VAS scores also improved postoperatively and did not differ significantly between the groups.

**Table 2:** Clinical outcomes in the Os subfibulare removed and not removed groups

Variable	Total (*n* = 44)	OSF removed (*n* = 23)	OSF not removed (c21)	*p*-value
PRE AOFAS	70 (55.75–77.5)	70 (59.5–78)	70 (51–77)	0.9718
POM 6 m AOFAS	86 (80.75–90)	89 (84.5–90)	85 (79–89)	0.0559
POY 1 year AOFAS	95.5 (90–100)	100 (92.5–100)	92 (90–100)	0.1449
PRE FAOS	43 (42–45)	42 (42–45)	44 (43–45)	0.1516
POM 6 m FAOS	60 (47–80.25)	60 (47–78)	55 (49–81)	> 0.999
POY 1 year FAOS	123 (110.75–147.5)	124 (113–154.5)	122 (110–137)	0.5568
PRE VAS	5 (4–6)	5 (4–6)	5 (4–6)	0.5492
POM 6 m VAS	2 (2–3)	2 (2–3)	2 (2–4)	0.3103
POY 1 year VAS	0.5 (0–2)	0 (0–1)	1 (0–2)	0.0716

Values are presented as mean (range)

OSF: os subfibulare, AOFAS: American Orthopaedic Foot and Ankle Society, FAOS: Foot and Ankle Outcome Score, PRE: preoperative, POM: postoperative month, POY: postoperative year, VAS: visual analog scale

Table 3 compares the radiological outcomes between subgroups. There were no differences in radiological outcomes between groups.

**Table 3:** Radiological outcomes in the Os subfibulare removed and not removed groups

Variable	Total (*n* = 44)	OSF removed (*n* = 23)	OSF not removed (*n* = 21)	*p*-value
PRE TTA (°)	8.75 (5.28–11.3)	9.9 (6.75–12.15)	7.9 (4.4–9.5)	0.1484
POM 6 TTA (°)	5.65 (4.18–7.92)	5.3 (4.3–8.16)	6 (4.1–7.45)	0.5335
POY 1 TTA (°)	4.63 (3.1–6.55)	4.7 (3.65–6.2)	4.56 (2.7–7.1)	0.9438
PRE ADT (mm)	6.64 (5.44–7.81)	6.75 (5.53–7.92)	6.63 (5.29–7.66)	0.7624
POM 6 ADT (mm)	5.62 (4.93–6.74)	5.89 (4.74–6.88)	5.33 (5.15–6.49)	0.8325
POY 1 ADT (mm)	5.15 (4.23–6.34)	5.29 (4.15–6.35)	5.02 (4.37–6.11)	0.8972

Values are presented as mean (range)

OSF: os subfibulare, ADT: anterior drawer test, TTA: talar tilt angle, PRE: preoperative, POM: postoperative month, POY: postoperative year

The concordance correlation coefficient (CCC) for the interobserver and intraobserver reliability of all radiological measurements were acceptable (Table 4, Supplementary Table 1) [19–21]. 

**Table 4:** Intraobserver concordance correlation coefficient for radiologic outcome measurements

Variable	Observer 1				Observer 2				Observer 3		
	CCC	95% LCI	95% UCI		CCC	95% LCI	95% UCI		CCC	95% LCI	95% UCI
TTA											
Preoperative	0.9933	0.9877	0.9963		0.9933	0.9877	0.9963		0.9942	0.9894	0.9968
6 months F/U	0.8724	0.7779	0.9283		0.9866	0.9756	0.9927		0.9934	0.988	0.9964
1 year F/U	0.9911	0.9832	0.9953		0.9958	0.9923	0.9977		0.9974	0.9933	0.9988
ADT											
Preoperative	0.9551	0.9193	0.9753		0.9429	0.8977	0.9685		0.9251	0.8557	0.9602
6 months F/U	0.8877	0.7324	0.9466		0.9494	0.9093	0.972		0.9783	0.9606	0.9881
1 year F/U	0.9338	0.8745	0.9645		0.9681	0.9424	0.9824		0.9597	0.9092	0.9802

CCC: concordance correlation coefficient, LCI: lower confidence interval, UCI: upper confidence interval, TTA: talar tilt angle, ADT: anterior drawer test, F/U: follow-up

## Discussion

The most important findings of the present research is that the groups in which ossicles were and were not removed had similar good results in terms of the FAOS, AOFAS, and VAS scores, and TTA, and ADT on stress radiographs at the final follow-up. Furthermore, there were no significant differences with regard to gender, age, and side of injury between the groups. Both the intra- and interobserver agreement on the radiological measurements were excellent. These findings suggest that removal of a small subfibular ossicle during the modified Broström procedure does not significantly affect clinical outcomes.

The occurrence of malleolar accessory ossicles in the general population ranges from 1 to 5.2% and they typically remain asymptomatic [22, 23]. In CLAI patients, however, ossicles found in this area are less likely to be developmental and are more likely to be associated with acute or chronic trauma [24]. The reported incidence of malleolar accessory ossicles in CLAI patients ranges from 34 to 66% [25, 26]. Determining the exact etiology of a malleolar accessory ossicle can be challenging [27]. 

Anatomic repair of the lateral ankle ligaments using the modified Broström technique achieves good or excellent results in 87–100% of patients [2, 27, 28]. In cases involving patients with a subfibular ossicle, the ossicle is often excised in individuals experiencing tenderness in the subfibular area [29]. With recent advances in and the widespread adoption of ankle arthroscopy, several studies have had successful outcomes with arthroscopic excision of malleolar ossicles [12, 18, 30]. However, despite careful removal of the ossicles in this procedure, it is often difficult to avoid cutting through ligament fibers. Being attached to the ossicle, the ATFL plays a crucial role in maintaining ankle joint stability [31]. Given the benefits and drawbacks associated with various treatment options, the appropriate surgical approach for subfibular ossicles remains uncertain. Hasegawa et al. [13] noted that these ossicles are often partially or completely embedded within the fibers of the AFTL. Consequently, removing an ossicle may result in the loss of remnant ligamentous tissue and create a gap within the AFTL that cannot be approximated adequately, leading to compromised anteroposterior stability.

Based on our findings, we concluded that removing an OSF ≤ 5 mm during the MBO did not have a significant impact on the outcomes. Considering the potential risks associated with OSF removal, it may not be necessary to remove an OSF ≤ 5 mm. Of course, regardless of size, if the patient complains of discomfort and the cause is presumed to be OSF, it is best to attempt removal.

This study has several limitations. First, the patients were followed for 1 year. Patients whose symptoms improved postoperatively were often lost to longer follow-up. Therefore, it was unknown about the occurrence of complications that could be detected during long-term observation. Additionally, this study has limitations due to its retrospective nature. Lastly, the study was conducted at a single institution with a relatively small number of subjects. Therefore, additional variables such as comorbidities could not be confirmed. Future research on more patients at multiple centers is necessary to determine the generalizability of the results and biomechanical study.

## Conclusions

When performing arthroscopic MBO on patients with CLAI, OSF ≤ 5 mm removal did not alter clinical or radiological outcomes, suggesting that excision may not be needed in asymptomatic patients. Considering the risks of the removal process, leaving it alone may be a treatment option.

## Electronic supplementary material

Below is the link to the electronic supplementary material.

Supplementary Material 1

## Data Availability

The datasets generated during and / or analysed during the current study are available from the corresponding author on reasonable request.

## Abbreviations

OSF: Os Subfibulare
CLAI: Chronic Lateral Ankle Instability
MBO: Modified Broström Operation
ATFL: Anterior Talofibular Ligament
CFL: Calcaneofibular Ligament
VAS: Visual Analog Scale
FAOS: Foot and Ankle Outcome Score
AOFAS: American Orthopaedic Foot and Ankle Society
ADT: Anterior Drawer Test
TTA: Talar Tilt Angle
IRB: Institutional Review Board
PACS: Picture Archiving and Communication System
CCC: Concordance Correlation Coefficient
