# Supplementary material for: Is it necessary to remove small fibular ossicles during an arthroscopic modified Broström operation for chronic lateral ankle instability?
Source: BMC Musculoskelet Disord. 2025 Apr 21;26:383. doi: 10.1186/s12891-025-08546-7 (PMC12013082; doi:10.1186/s12891-025-08546-7)
Supplement: Supplementary file 1 — Supplementary Material 1 [file 12891_2025_8546_MOESM1_ESM.docx]

**SUPPLEMENTARY MATERIAL**

**Supplementary Table 1.** Interobserver Concordance Correlation Coefficient for radiologic outcome measurements

| **Variable** | **CCC** | **95% LCI** | **95% UCI** |
| --- | --- | --- | --- |
| TTA |  |  |  |
| Preoperative | 0.9983 | 0.9973 | 0.9990 |
| 6 months F/U | 0.9968 | 0.9947 | 0.9981 |
| 1 year F/U | 0.9965 | 0.9942 | 0.9980 |
| ADT |  |  |  |
| Preoperative | 0.9847 | 0.9747 | 0.9911 |
| 6 months F/U | 0.9821 | 0.9705 | 0.9896 |
| 1 year F/U | 0.9895 | 0.9826 | 0.9939 |

CCC: concordance correlation coefficient, LCI: lower confidence interval, UCI: upper confidence interval, TTA: talar tilt angle, ADT: anterior drawer test, F/U: follow-up.
